# Antidepressants in the Treatment of Functional Dyspepsia: A Systematic Review and Meta-Analysis

**DOI:** 10.1371/journal.pone.0157798

**Published:** 2016-06-16

**Authors:** Yaoyao Lu, Meng Chen, Zhiyin Huang, Chengwei Tang

**Affiliations:** Department of Gastroenterology, West China Hospital, Sichuan University, Chengdu, Sichuan, China; University Hospital Llandough, UNITED KINGDOM

## Abstract

**Background:**

Antidepressants have been empirically used in the treatment of functional dyspepsia (FD). However, results from recent clinical trials investigating their efficacy are conflicting. The aim of this study is to evaluate the efficacy of antidepressants in the management of FD in adults.

**Methods:**

Databases of MEDLINE, EMBASE, the Cochrane Central Register of Controlled Trials and BIOSIS Previews were searched for all randomized controlled trials (RCT) investigating efficacy of antidepressants in the management of FD in adult patients. Data of overall symptom unimproved and adverse events were compared between the antidepressants and placebo group.

**Results:**

The search strategy identified 432 citations. Of those, eight RCTs met the inclusion criteria and were included in the meta-analysis. The pooled relative risk (RR) of symptom unimproved with tricyclic antidepressants (TCAs) versus placebo was 0.76 (95% CI: 0.62 to 0.94, *P* = 0.01; I^2^ = 0%, *P* = 0.39). By contrast, selective serotonin reuptake inhibitors (SSRIs) did not show a benefit over placebo (RR = 1.00, 95% CI: 0.86 to 1.17, *P* = 0.95; I^2^ = 0%, *P* = 0.82). Adverse events were significantly more frequent among patients receiving antidepressants than those receiving placebos (RR = 1.64, 95% CI: 1.14 to 2.35, *P* = 0.007).

**Conclusion:**

TCAs but not SSRIs, are effective in the treatment of FD, but antidepressants were also associated with more adverse events compared with placebo.

## Introduction

Functional dyspepsia (FD) is a gastrointestinal disorder defined as the experience of postprandial fullness, early satiation, epigastric pain or burning originating in the gastroduodenal in the absence of any organic disease that could explain the symptoms [1]. FD accounts for a large percentage of gastroenterology clinical visits, and its prevalence can reach up to 15.7% in the general population [2–5]. Despite its favorable prognosis, FD can impair suffers’ quality of life, and the condition places a strain on health resources because patients with FD tend to seek excessive medical care [4, 6–8]. Proton pump inhibitors (PPIs) and prokinetic agents are currently recommended as first-line treatment for FD [9]. However, the efficacy of these medicines was limited. According to one meta-analysis, symptom relief occurs for only 40.3% of FD patients receiving PPIs, compared with 32.7% receiving placebo [10]. The treatment efficacy of prokinetic agents is similar with that of PPIs [11].

Antidepressants are empirically used in the treatment of FD and show promising efficacy. They may alleviate symptoms of FD through treatment of comorbid psychological diseases, enhancing gastric accommodation and manipulating pain perception. Psychosocial and psychiatric factors may play an important role in the pathogenesis of FD. Population-based studies have demonstrated that, compared with healthy controls, FD patients may have higher levels of depression before diagnosis, are more likely to suffer from co-morbid anxiety and are more depressed over longer-term follow-up periods [2, 3]. Psychological abuse is associated with pain symptoms in FD [12]. Besides, impairment of gastric accommodation is present in about 40% of FD cases and is well correlated with certain symptoms [13]. Some antidepressants can enhance meal-induced gastric relaxation and alleviate related symptoms [14–16]. Additionally, epigastric pain is one of the main symptoms of FD, and visceral hypersensitivity to acid and distension may contribute to its development. Antidepressants show beneficial effects in the treatment of such pain disorder as functional chest pain and chronic back pain [17, 18]. Previous studies have found that antidepressants are effective in treating patients with irritable bowel syndrome (IBS, another subset of functional gastrointestinal disorders) [19, 20] Since these two diseases share much in common, it is possible that antidepressants are effective for both diseases.

Despite their frequent use in clinical practice, the efficacy of antidepressants in the treatment of FD remains controversial. Three previous meta-analyses have been conducted on this issue [21–23], but all the three have methodological limitations. These studies also failed to investigate the efficacy of antidepressants in FD in a separated analysis or to include the recently published randomized controlled trials (RCTs). Therefore, we offer an updated meta-analysis considering treatment efficacy and tolerability of antidepressants in adult patients with FD.

## Methods

### Search strategy and inclusion criteria

The meta-analysis was conducted according to the Preferred Reporting Items for Systematic Reviews and Meta-analysis (PRISMA) statement [24]. The databases of MEDLINE (from 1946), EMBASE (from 1974), the Cochrane Central Register of Controlled Trials and BIOSIS Previews (from 2001 to 2012) were searched to December 2015 for all studies that examine the efficacy of antidepressants in the treatment of FD in adult patients. The search terms were as follows: functional dyspepsia, epigastric pain syndrome, postprandial distress syndrome, antidepressive agent*, antidepressant*, imipramine, clomipramine, trimipramine, lofepramine, desipramine, fluvoxamine, amoxapine, amitriptyline, nortriptyline, maprotiline, protriptyline, sertraline, mianserin, setiptiline, fluoxetine, paroxetine, milnacipran, trazodone, venlafaxine, mirtazapine, bupropion, citalopram, escitalopram, doxepin, isocarboxazid, nefazodone, phenelzine, tranylcypromine, zimelidine, dothiepin, dosulepin and flupentixol. References from reviews and eligible studies were also retrieved in order to identify additional potentially relevant studies. The search was repeated on February 22, 2016 and no new trail was identified.

Two reviewers independently screened the original search results for eligible studies. Inclusion criteria were as follows: i) RCTs that compared the efficacy of antidepressants with placebos among adult FD patients; and ii) studies reported data of symptom unimprovemd or adverse events with an abstract or full text present. There was no language restriction for the studies. Crossover studies of high-quality were also included. In the case of eligible studies with incomplete data, we attempted to contact the corresponding authors to obtain further information.

### Data extraction

Two of the authors independently extracted data, which included the first author, study design, publication year, inclusion criteria, sample size, types and doses of antidepressants, treatment and follow-up duration, total numbers of patients with overall symptom unimproved and adverse events rates. Discrepancies were resolved through discussion. Data were extracted from an intention-to-treat (ITT) analysis when possible, with a per-protocol analysis used as the alternative. The quality of each eligible study was also assessed using the Jadad scale, which is widely employed to assess the quality of RCTs [25]. Studies with Jadad score of at least 3 are considered of moderate to high quality.

### Data synthesis and statistical analysis

We conducted the meta-analysis using Review Manager v5.0 (Cochrane Collaboration, Oxford, UK). Data on overall symptom unimprovemed and adverse events were compared between the antidepressant and placebo groups. A subgroup analysis concerning treatment efficacy of each class of antidepressants separately was also conducted to determine whether different classes of antidepressants had different effects. The pooled statistics were reported as relative risks (RRs) with 95% confidence intervals (95% CIs). The number needed to treat (NNT) and number needed to harm (NNH) were also calculated when there were significant differences between patients taking antidepressants and the placebo group. The data was pooled using a random effect model in order to obtain a conservative estimate of treatment efficacy. Cochrane Q test, the result of which was presented as I^2^, was also performed to assess the heterogeneity between the studies. When the Cochrane Q test showed obvious heterogeneity between studies (I^2^ ≥ 50%), a sensitivity analysis was conducted to explore the possible origin of the heterogeneity. Funnel plot was used to test publication bias. The statistical methods of this study were reviewed by Professor Guangyun Mao from Center on Clinical & Epidemiological Research, Affiliated Eye Hospital of Wenzhou Medical University.

## Results

### Characteristics of studies included in the meta-analysis

**Fig 1:**
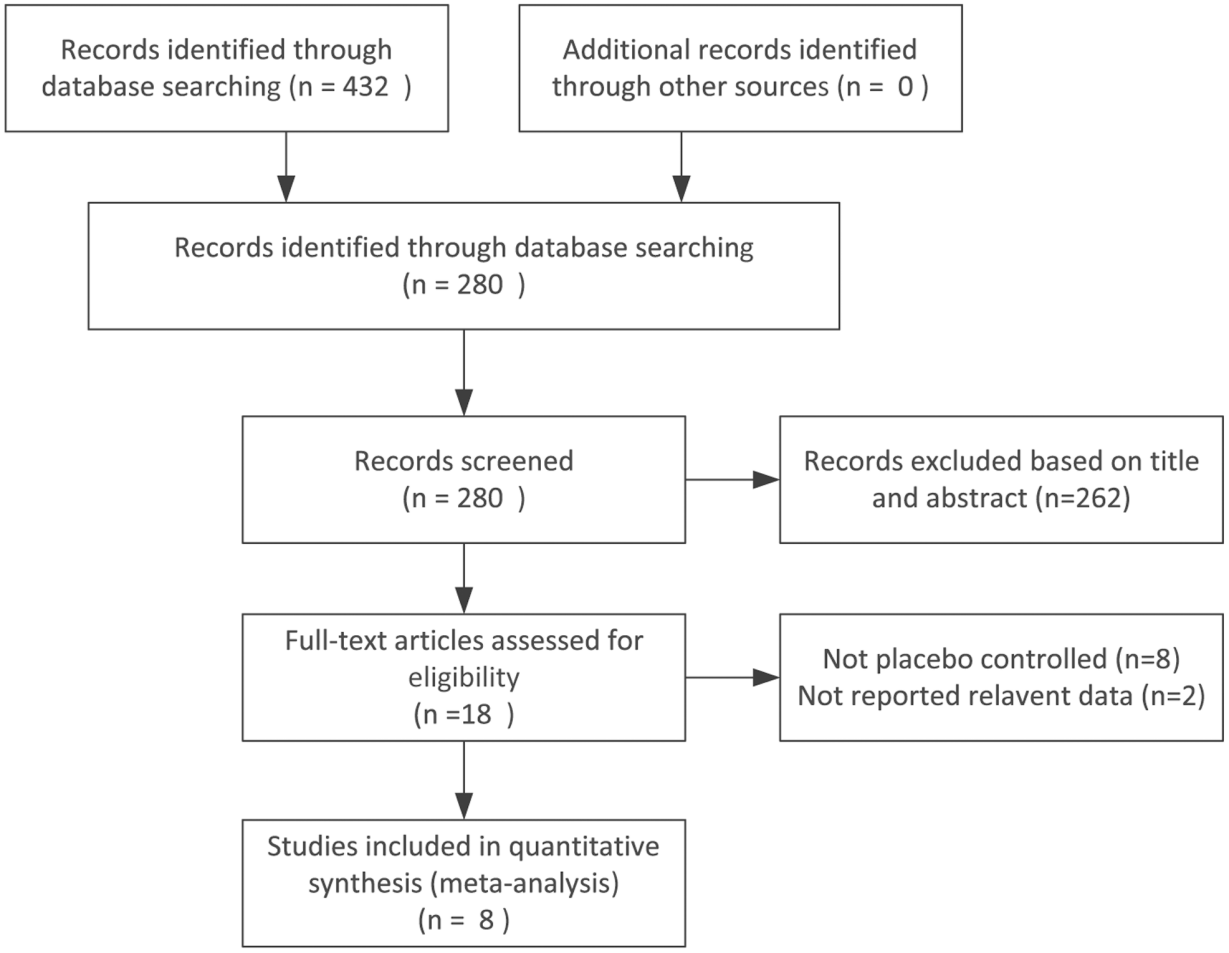
Identification of eligible studies.

The search strategy generated 432 citations, including eighteen RCTs that investigated the treatment efficacy of antidepressants in FD [26–43]. Ten of these eighteen trials compared antidepressants with placebos, seven compared antidepressants + “conventional treatment” with “conventional treatment” and one compared antidepressants with no treatment. Another two potentially eligible studies were excluded from the meta-analysis for failure to reporting relevant data. Therefore, eight clinical trials [26–33], including six RCTs and two cross-over studies, were ultimately included. All of the included trails were written in English. A flow chart of the selection process is shown in Fig 1.

Among the included studies, six were published in full text and two were in abstract form only [27, 29]. The antidepressants investigated in the eight studies included were sertraline, imipramine, amitriptyline, venlafaxine, nortriptyline and melitracen + flupenthixol (M + F). All studies but one reported ITT analysis results [28]; for that study, we used per-protocol analysis results. All eight articles were of moderate to high quality with Jadad scores of at leat 3. Detailed information is shown in Table 1. The corresponding authors of three studies were contacted by e-mail in an effort to obtain unpublished data needed for the present study, but no responses were received [28, 30, 31].

**Table 1:** Characteristics of clinical trials examining antidepressants in functional dyspepsia.

Reference	Study design	Jadad score	Inclusion criteria	Sample size	Treatment; length of follow-up	Response to treatment	Adverse effect
						Antidepressant vs. Control	Antidepressant vs. Control
Talley NJ 2015 [26]	RCT	4	Met the Rome II criteria; PPI treatment failed; without depression; ages 18–75 y.	292	Amitriptyline 50 mg or escitalopram 10 mg or placebo qd for 12 w; 9 m	45.6%* vs. 40%	29.2%# vs. 21%
Kaosombatwattana U 2015 [27]	RCT	3	FD patients; PPI and/or prokinetics treatment failed	61	Nortriptyline 10 mg or placebo qd for 8 w.; NR	53.6% vs,54.5%	NR
Tan VP 2012 [28]	RCT	5	Met the Rome II criteria; Hp-; ages 17–71 y	193	Sertraline 50 mg or placebo qd for 8 w; 8 w	28.4% vs.27.6%	NR
Wu JC 2011 [29]	RCT	3	Met the Rome II criteria; PPI treatment failed; ages > 18 y	107	Imipramine 50 mg or placebo qd for 12 w; 12 w	63.6% vs.44.2%	NR
Braak B 2011 [30]	RCT	5	Met the Rome III criteria; PPI treatment failed; without depression; ages 18–65 y	38	Amitriptyline 25 mg or placebo qd for 8 w; 8 w	No differences between the two groups;	72% vs.35%
Van Kerkhoven LA 2008 [31]	RCT	3	Clinical judgement; ages > 18 y	160	Venlafaxine (75 mg qd 2 w—150 mg qd 4 w—75 mg qd 2 w) or placebo for 8 w; 20 w	37% vs. 39%	NR
Hashash JG 2008 [32]	Crossover study	4	Met the Rome III criteria; PPI treatment failed; Hp eradication; without depression; ages 18–65 y	25	M + F or placebo 1 tablet bid for 4 w; 2 weeks for wash-out; 6 w	70.8% vs.24%	20% vs. 8%
Mertz H 1998 [33]	Crossover study	3	FD with sleep disorder;Hp-; wihtout depression; ages 20–65 y	7	Amitriptyline 50 mg qn or placebo for 8 w; 3 w for wash-out; 11 w	71% vs. 28%	NR

RCT: randomized controlled trial. NR: not reported. IBS: irritable bowel syndrome. FD: functional dyspepsia. PPI: proton pump inhibitor. qd: once per day. qn: once every night. M + F: melitracen+flupenthixol

*53% in the amitriptyline group vs 38% in the escitalopram group.

^#^30% in the amitriptyline group vs 29% in the escitalopram group.

### Treatment efficacy

**Fig 2:**
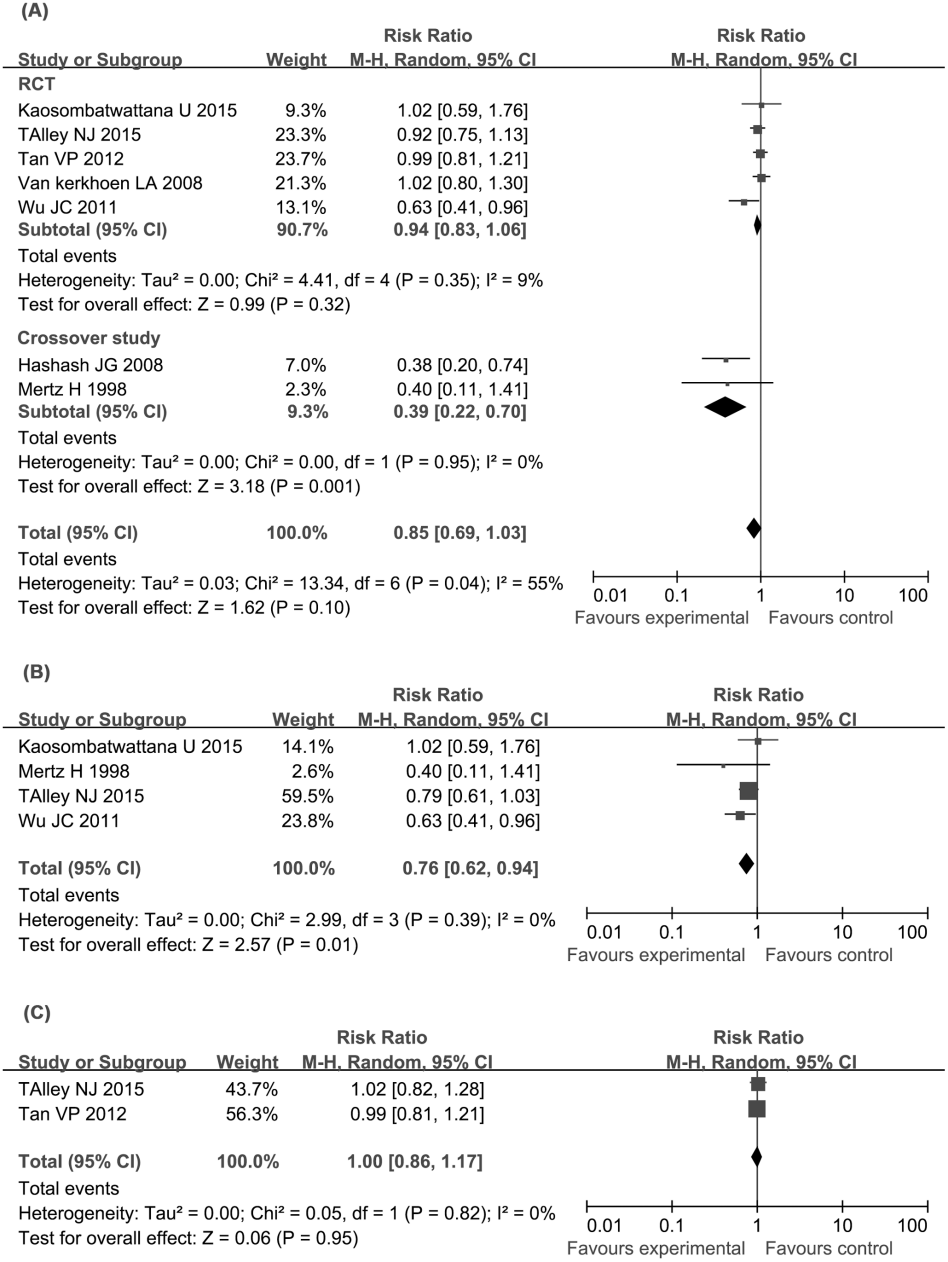
Forest plot showing efficacy of antidepressants versus placebo. (A) Treatment efficacy of all antidepressants investigated. (B) Treatment efficacy of tricyclic antidepressants (TCAs). TCAs significantly reduce risk of symptom unimproved. (C) Treatment efficacy of selective serotonin reuptake inhibitors (SSRIs). SSRIs do not show a benefit over placebo.

Seven of the eight RCTs reported the overall symptom improvement rates in response to treatment. Of these, three studies reported the exact symptom scores in each group, and three studies reported on quality-of-life improvements. In total, 252 of 463 patients reported that symptoms were unimproved in the antidepressant group, compared with 139 of 370 patients in the placebo group, with no significant difference between the two groups (RR = 0.85, 95% CI: 0.69 to 1.03, *P* = 0.10). The Cochran Q test showed a moderate level of heterogeneity between the studies (I^2^ = 55%, *P* = 0.04; Fig 2A). In the sensitivity analysis according to the study design, no heterogeneity was found when the crossover studies were removed from the analysis, and still, administration of antidpressants did not show a benefit compared with placebo (RR = 0.94, 95% CI, 0.83 to 1.06; Fig 2A). In subgroup analysis, administration of tricyclic antidepressants significantly associated with reduced number of patients with symptom unimproved compared with placebo (RR = 0.76, 95% CI: 0.62 to 0.94; I^2^ = 0%, *P* = 0.39;) (Fig 2B) with an NNT of 7 (95% CI: 4, 26). However, no significantly differences were found between selective serotonin reuptake inhibitors (SSRIs) group and placebo group (RR = 1.00, 95% CI: 0.86 to 1.17; I^2^ = 0%, *P* = 0.82; Fig 2C).

### Adverse effects

**Fig 3:**
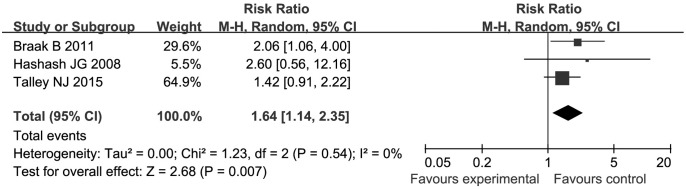
Forest plot showing adverse effects of antidepressants versus placebo. Antidepressants significantly increase adverse events compared with placebo.

Three of the eight studies, which included 379 patients, reported the exact number of adverse events. A total of 75 (31.6%) patients in the antidepressant group compared with 29 (20.4%) in the placebo group were reported to have experienced adverse events. A significant association was found between the use of antidepressants and an increase in adverse events (RR = 1.64, 95% CI: 1.14 to 2.35, *P* = 0.007) with an NNH of 9 (95% CI: 5, 44) and no heterogeneity was found among the included studies (I^2^ = 0%, *P* = 0.54) (Fig 3). No serious adverse event was reported in any study.

### Publication bias

**Fig 4:**
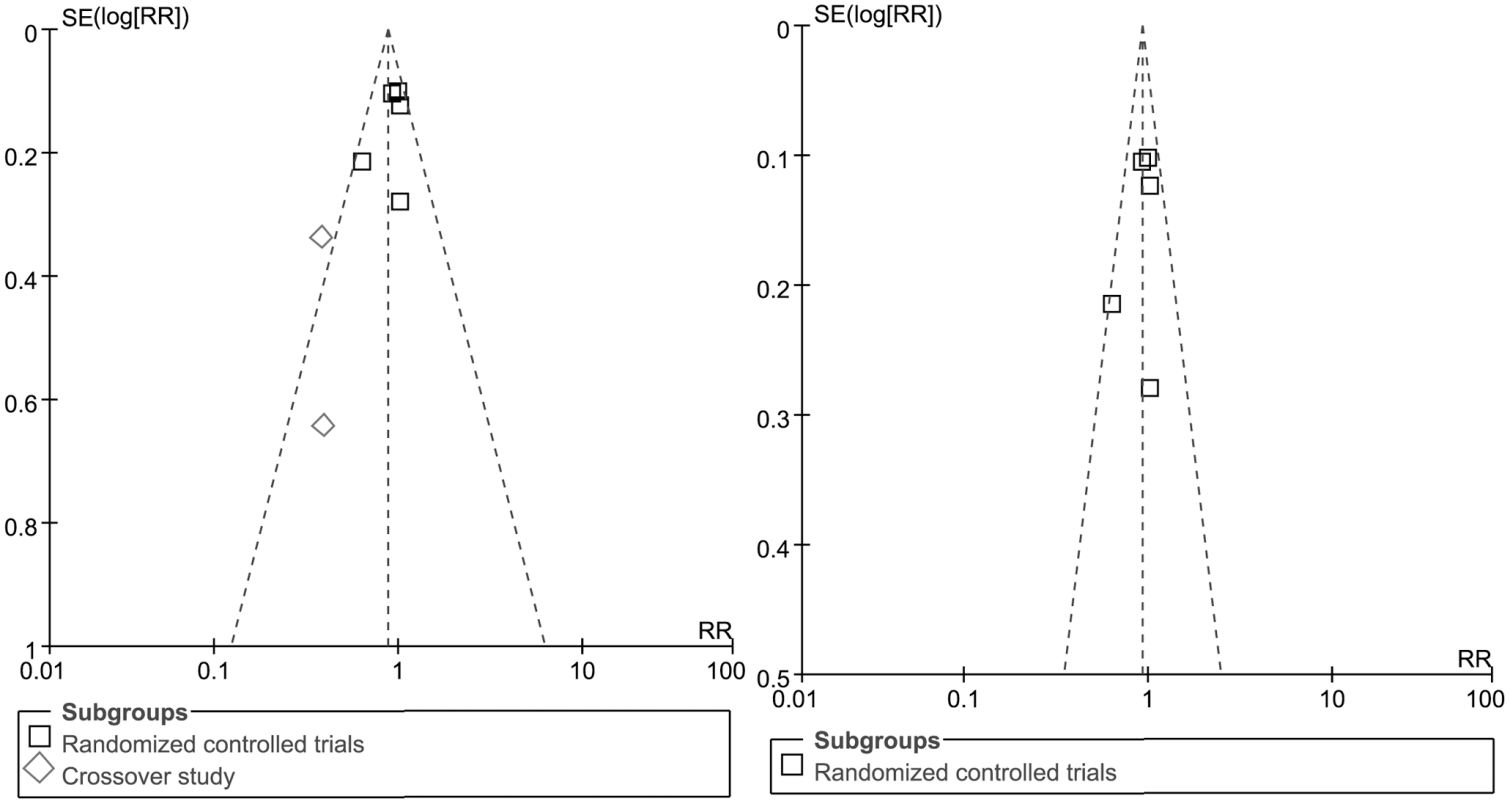
Funnel plot of clinical trials of antidepressants versus placebo. (A) Funnel plot of all included trials. (B) Funnel plot of randomized controlled trials with no obvious publication bias found.

The funnel plot regarding the treatment efficcacy of all included trials showed asymmetry (Fig 4A). However, the funnel plot of RCTs included seems asymmetrical which means publication bias is unlikely among these studies (Fig 4B).

### Antidepressants plus “conventional treatment” compared with “conventional treatment” alone

The quality of studies comparing efficacy of antidepressants + “conventional treatment” with “convention treatment” alone was poor. Six of the seven studies investigated treatment efficacy of M + F (deanxit). Detailed information is shown in S1 Table. Results from these studies were also pooled. The RR for symptom unimproved with antidepressants + “conventional treatment” versus “conventional treatment” alone was 0.3 (95% CI: 0.22 to 0.42; I^2^ = 0%, *P* = 0.92) (S1 Fig) with an NNT of 4 (95% CI: 3 to 5). Adverse events rates were equivalent among patients allocated to either of the two groups (RR = 1.72, 95%CI: 0.69 to 4.32; I^2^ = 35%, *P* = 0.19) (S2 Fig).

## Discussion

A meta-analysis of RCTs that assessed the efficacy of antidepressants in managing adult FD was conducted. TCAs, but not SSRIs, are effective in the treatment of adult FD. Patients allocated to the antidepressants group reported more adverse events than those allocated to placebo group, with an NNH of 9. Antidepressants used in combination with “conventional treatment” also showed increased symptom improvement rates compared with “conventional treatment” alone. However, studies investigating this issue are of low quality.

The main findings of this meta-analysis appear to be inconsistent with previous studies. We identified 3 meta-analyses on this topic, all of which concluded that antidepressants are effective in relieving FD symptoms. However, all of these studies were methodologically different from ours. One study included both IBS and FD patients but did not analyse them separately [23]. The other two studies included patients who were treated with levosulpiride [21, 22]. Levosulpiride is a dopamine D2 receptor antagonist and a mild 5-HT4 receptor agonist, which make it both a prokinetic agent and an antidepressant [44, 45]. the inclusion of levosulpiride may have introduced bias because previous studies have found that prokinetic agents are effective in treating FD [46]. In addition, one of the reports pooled the results of studies that examine antidepressants compared with placebos, “conventional treatment” or no treatment into a single analysis [21]. Moreover, this same study assessed the therapeutic effect by comparing means of indices evaluating FD symptoms before and after treatment with antidepressants. As is known, a placebo response is common among FD patients, [47] for which reason it cannot be concluded that antidepressants are effective in the treatment of FD. Finally, none of these meta-analyses assessed the quality of the included studies.

In the present study, several methods were used to overcome these limitations. First of all, we included recently published RCTs which were not included in previous meta-analyses. Quality assessments were also conducted, and only moderate-to-high-quality, placebo-controlled studies were included in our meta-analysis. Owing to the small number of RCTs identified, we also included crossover studies. A sensitivity study was conducted when heterogeneity was present, and a subgroup analysis was conducted to evaluate the efficacy of different classes of medication. Data from an ITT analysis were used whenever possible, further ensuring that the treatment effect was not overestimated. The adverse effects of antidepressants were also pooled and assessed, steps that were not taken in previous studies.

There are limitations to the present meta-analysis, most of which are due to the characteristics of the included studies. First, only 6 RCTs were included, which is a relatively small number. In the subgroup analyses, only two and three studies were included in the assessment of the efficacy of SSRIs and the tolerability of antidepressants, respectively. However, according to the present results, the ineffectiveness of SSRIs is almost unlikely due to the small sample size. To include as many eligible studies as possible, we also contacted the corresponding authors of three RCTs in an effort to obtain unpublished data. Second, moderate heterogeneity was found when the crossover studies were included. This heterogeneity may have resulted from the study design, type of medicine used or treatment duration, among other factors. It is notable that a crossover study conducted by Hashash *et al*. has found a very large effect favouring the use of M + F [32]. Melitracen is a TCA, while flupenthixol is a typical antipsychotic agent. The combination of the two has both anxiolytic and antidepressant properties. However, no RCT that compares the efficacy of M + F with placebos in FD has been published to date. When the crossover studies are excluded from the analysis, both heterogeneity and publication bias disappeared. Third, although all of the included RCTs were of high to moderate quality, two articles were meeting abstracts that did not contain the full texts with additional information [27, 29]. Fourth, the diverse inclusion criteria used in each included article may have introduced bias.

The present study finds improvement in patients taking TCAs but not in those taking SSRIs. This difference may be due to the superior pain relieving function of TCAs. One meta-analysis found that TCAs, but not SSRIs, can significantly reduce functional chest pain [17]. An RCT by Talley *et al*. has found that patients with ulcer-like FD who take amitriptyline, a TCA, are more likely to report symptom improvement than patients with dysmotility-like FD [26], but improvement was not observed in patients taking escitalopram, a SSRI. It is possible that antidepressants are more effective in managing certain subtypes of FD, and this avenue should be explored in the future. In addition, the frequently reported adverse effects of antidepressants may limit their efficacy. In the present analysis, adverse events occurred significantly more frequently in the antidepressant group than in the placebo group with an NNH being 9. Fortunately, however, no serious adverse events were reported. Due to the small number of studies included, we did not analyse the tolerability of TCAs and SSRIs separately.

## Conclusions

In conclusion, the current data support the use of TCAs but not SSRIs in the management of FD in adults. However, the present data also indicate an increased risk of adverse events for antidepressants. Further well-designed, high-quality studies are needed, in particular to investigate the treatment efficacy of TCAs for subtypes of FD.

## Supporting Information

S1 Fig Forest plot showing the efficacy of antidepressants + “conventional treatment” versus “conventional treatment.” (EPS)

S2 Fig Forest plot showing adverse effects of antidepressants + “conventional treatment” versus “conventional treatment.” (EPS)

S1 File PRISMA Checklist. (DOC)

S1 Table Characteristics of studies investigating efficacy of antidepressants + “conventional treatment” versus “conventional treatment.” (DOC)
