# Supplementary material for: Antidepressants in the Treatment of Functional Dyspepsia: A Systematic Review and Meta-Analysis
Source: PLoS One. 2016 Jun 16;11(6):e0157798. doi: 10.1371/journal.pone.0157798 (PMC4911162; doi:10.1371/journal.pone.0157798)
Supplement: S1 Table — (DOC) [file pone.0157798.s004.doc]

S1 Table. Characteristics of studies investigating treatment efficacy of antidepressants + “conventional treatment” compared with “conventional treatment” in functional dyspepsias.

| study | Study design | Jaded score | Inclusion criteria | Sample size | Treatment; length of follow-up | Response to treatment | Side effect |
| --- | --- | --- | --- | --- | --- | --- | --- |
|  |  |  |  |  |  | Antidepressant vs. control | Antidepressant vs. control |
| Zhao W 2013 | RCT | 0 | Rome III criteria EPS; HAD: 8-15 | 58 | Esomeprazole 40 mg qd + Deanxits 1# bid vs. Esomeprazole 40 mg qd for 8w; 0 w. | 83.87% vs. 62.97%  26/31 vs 17/27 | 10/31 vs. 0/27 |
| Qiu Z 2013 | RCT | 1 | Roma Ⅲ criteria; severe functional dyspepsia | NR | rabeprazole 10 mg + mosapride 5 mg tid. + Deanxits 10.5 mg qd. vs. rabeprazole 10 mg + mosapride 5 mg tid. for 8w; 4w | 97.32% VS 17.98% in patients with mild to moderate anxiety and depression; no difference in remaining patients. | NR |
| Song JM 2011 | RCT | 1 | Rome III criteria; severe functional dyspepsia patients | 44 | Mirtazapine 30 mg qd + convential treatment vs. lansoprazole 30 mg qd. + itopride 50 mg tid. for NR w; NR. | 81.8% vs 13.6%  18/22 vs 3/22 | 4/22 vs 0/22  18.2% vs 0 |
| Li Z 2004 | RCT | 3 | Epigastric discomfort ≥4 w; HAMD > 7 and/or HAMD > 7 | 129 | Conventional treatment + sertraline 50 mg qd. vs. Famotidine 20 mg tid. + domperidone 5 mg tid. for 8 w; NR | 86% vs. 52%  56/65 vs. 33/64 | 3/65 vs 3/64 |
| Gao FY 2011 | RCT | 2 | Rome Ⅲ criteria | 120 | Itopride + Deanxits 2 # qd. vs. Itopride 50 mg tid. for 4 w; 6m. | 53/57 vs. 46/57 | 3/60 vs. 3/60 |
| Chen YC 2014 | RCT | 2 | Rome III criteria | 200 | Labeprazole 10 mg qd. + mosapride 5 mg tid. + Deanxits 1# qd vs. conventional treatment for 8 w; 4 w | 77/84 vs. 58/83 | 10/100 vs. 8/100 |
| Guo QX 2015 | RCT | 2 | Clinical judgement. | 110 | Deanxits + conventional treatment vs. conventional treatment for NR w; NR | 85.45% vs 54.55%  47/55 vs 30/55 | NR |

NR: Not reported. RCT: Randomized controlled trial. EPS: Epigastric pain syndrome; HADS: Hospital anxiety and depression scale; HAMA: Hamilton anxiety scale; HAMD: Hamilton depression scale.
